# The triterpenoid curcumene mediates the relative hydrophilicity of *Bacillus subtilis* spores

**DOI:** 10.1128/mbio.03024-24

**Published:** 2024-11-29

**Authors:** Stefany Castaldi, Giuliana Donadio, Ivana Staiano, Ezio Ricca, Rachele Isticato

**Affiliations:** 1 Department of Biology, Complesso Universitario Monte S. Angelo, University of Naples Federico II, Naples, Italy; 2 NBFC, National Biodiversity Future Center, Palermo, Italy; National Institute of Child Health and Human Development (NICHD), Bethesda, Maryland, USA

**Keywords:** *Bacillus subtilis*, spore coat, sporulation, spores

## Abstract

Spores of *Bacillus subtilis* are surrounded and protected by the coat and the crust, multi-layered structures mainly made of proteins and polysaccharides. These polysaccharides are covalently linked to some of the coat and crust proteins and influence some spore properties, such as surface adhesion and hydrophilicity. This study reports that a mutant strain lacking the *spsA-L* operon, encoding 11 enzymes involved in the synthesis of spore surface polysaccharides, produced spores exposing on their surface hydrophobic molecules that were responsible for the drastic reduction of hydrophilicity of the mutant spores. Biochemical and genetic data support the identification of the C_35_-terpenoid curcumene, a precursor of the spore-associated lipid sporulene, as the highly hydrophobic molecule present on the surface of mutant spores.

**IMPORTANCE:**

Bacterial spores are the most resistant cell forms on Earth. The metabolically quiescent spores withstand conditions that would be lethal for other cells, maintaining the capacity to sense the environment and respond to the presence of favorable conditions by germinating. Such remarkable resistance is also due to the complex layers that surround the spore cytoplasm and protect it against damaging factors. Altogether, the spore surface layers form a complex cell structure composed of proteins, polysaccharides, and, as highlighted by this study, also of lipids. Understanding the complexity of the spore surface and the specific molecules involved in its structure is an essential step for unraveling the mechanisms underlying the spore’s resistance to environmental assaults.

## INTRODUCTION

*Bacillus subtilis* is the model system for bacterial spore formers, and studies on this organism have contributed to clarifying the structure of bacterial (endo)spores and the processes of spore formation and germination. When cell growth is no longer allowed by nutrient starvation or other unfavorable environmental conditions, some stationary cells enter the irreversible program of spore formation (1–4). An asymmetric cell division occurs, generating a large mother cell and a small forespore that, once matured into a spore, is released into the environment by the autolysis of the mother cell (1–4). Once released, the metabolically quiescent spore can persist indefinitely in the absence of water and nutrients, surviving extremes of heat and pH, the presence of UV radiation, solvents, hydrogen peroxide, and lytic enzymes (1–4). Although metabolically quiescent, the spore responds to the presence of water, nutrients, and favorable environmental conditions, germinating and thus generating a cell able to grow and, eventually, to resporulate. The processes of sporulation and germination have been extensively reviewed previously (1–5).

The resistance at conditions that would be lethal for vegetative cells is mainly due to the peculiar structure of the spore. The spore cytoplasm (core) is characterized by a low water content and is surrounded by various protective layers, a peptidoglycan-like cortex, a multilayered coat, and the outermost crust (6–8). The proteins forming the various coat layers and the crust are all produced in the mother cell cytoplasm and deposited in an ordered manner around the forming spore (6–8). The outermost layer, the crust, is composed of at least six proteins: CotY, the major structural crust component; CotV and CotX, structural components also suggested as targets of glycosylation; CotZ, the main regulator of the crust assembly; CotW, necessary for the maintenance of crust structure (9–13); and CgeA, unambiguously identified as a glycoprotein required to initiate the assembly of polysaccharides to the spore surface (14). In addition, coat and crust are also composed of polysaccharides produced in the mother cell during sporulation and covalently linked to some coat and crust proteins (12, 14, 15). These polysaccharides modulate the relative hydrophilicity of the spore (6–8) and are known to contain rhamnose, galactose, quinovose, glucosamine, and muramic lactam residues, but their structure is still not known (16, 17). The products of several genes participate in the synthesis of spore-associated polysaccharides (13, 15, 17, 18). Mutations in the *yfnHGFED* operon affect the crust structure and expand the localization of the surface glycans (13, 15), whereas mutations in the *cgeD* gene of the *cgeAB-cgeCDE* operons also expand the surface glycans but cause a reduced hydrophilicity of the spore (15). Less hydrophilic spores are also produced by null mutations in the *spsM* gene and/or in the eleven-gene operon *spsA-L* (18). The promoter-proximal genes of the operon, *spsAB,* code for two glycosyltransferases (GT) whose specific substrates and products are not known even if one of them, SpsA, has been structurally characterized in detail and shown to be a nucleotide-sugar-dependent GT, belonging to the enzyme family GT-2 (19, 20). The last four genes of the operon, *spsIJKL*, code for the enzymes required for the biosynthesis of rhamnose (21), whereas *spsCDEFG* and the single gene *spsM* are required for the synthesis and transfer to the spore surface of legionaminic acid (22), a nine-carbon monosaccharide found in glycoconjugates present on the cell surface of some bacteria, including the pathogens *Campylobacter jejuni*, *Acinetobacter baumanii,* and *Legionella pneumophila* (23).

Previous studies have shown that mutant strains lacking the *spsA-L* operon (17, 18) produce altered spores, much less hydrophilic than their isogenic wild-type. Such mutant spores in an aqueous solution form large aggregates that tend to precipitate (18). Similar features were observed in spores of strains lacking the *cotXYZ* operon or the *cotW* gene (12), whereas only a limited reduction of hydrophilicity was detected when only the CotV protein was not produced (12).

In this context, we show that when the carbohydrates synthesized by the SpsA-L enzymes are not produced, hydrophobic molecules are exposed on the surface of the mutant spore, which, as a consequence, become hydrophobic and form aggregates in aqueous environments. The triterpenoid curcumene (24, 25) was identified as the molecule responsible for the hydrophobicity of *spsA-L* mutant spores.

## RESULTS

### The SpsA-L proteins contribute to the spore integrity

**Fig 1:**
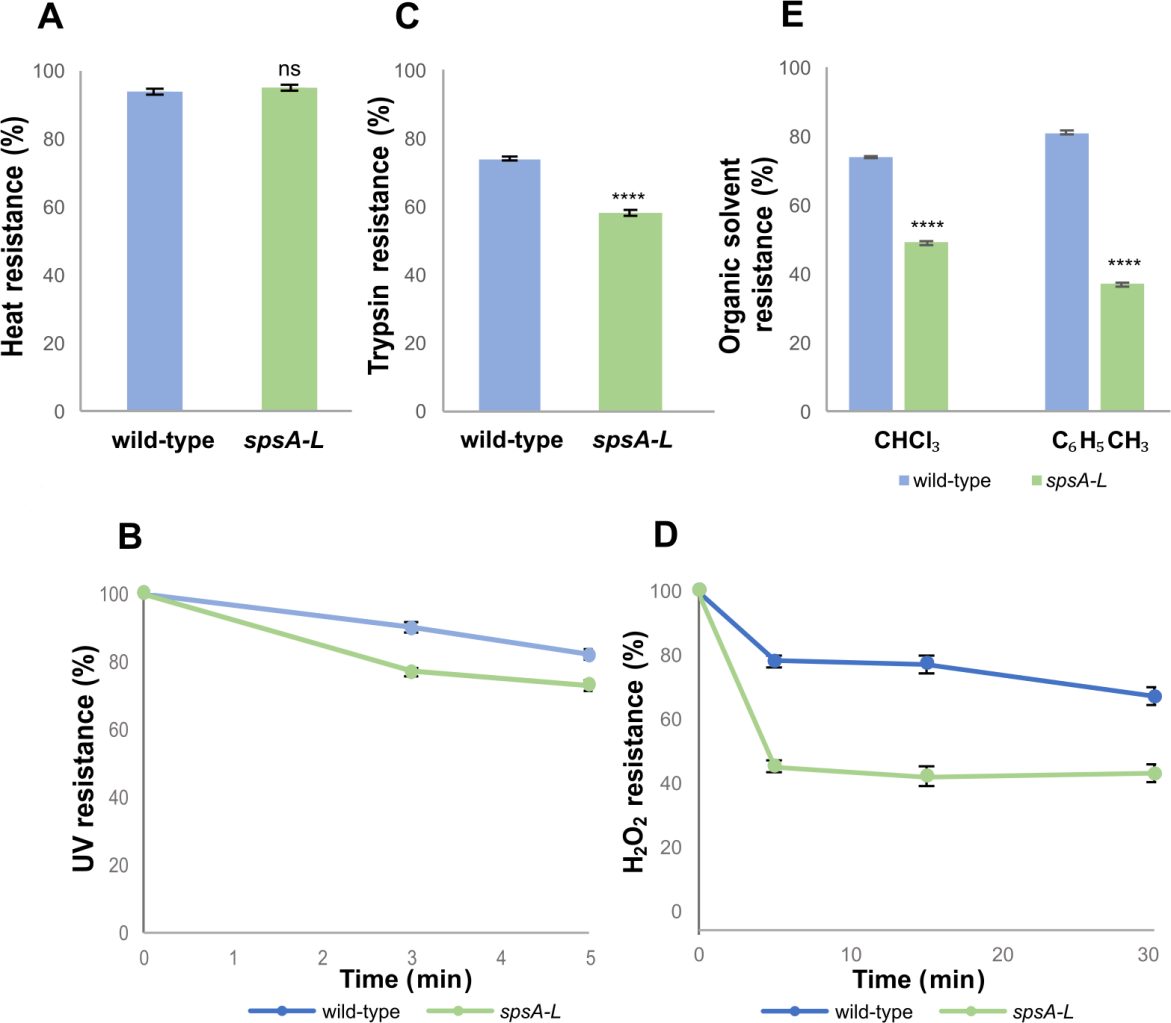
Evaluation of *spsA-L* mutant spores resistance. Purified spores (1.5 ± 0.1 × 10^8^) of wild-type and isogenic *spsA-L* mutants were compared for their resistance properties by assessing the viability (CFU) on LB agar plates. (A) Heat resistance: 80°C for 10 min. (B) Ultraviolet (UV) light resistance: room temperature for 3 and 5 min under a UV lamp (700 erg/mm^2^). (C) Trypsin resistance: 0.1 mg/mL trypsin for 1 h at 37°C. (D) Hydrogen peroxide (H_2_O_2_) resistance: H_2_O_2_ 0.036% at room temperature for 5, 15, and 30 min. (E) Organic solvent resistance: chloroform (CHCl_3_) or toluene (C_6_H_5_CH_3_) (Sigma-Aldrich) for 10 min at room temperature. The data are averages from three independent experiments performed with spores prepared independently. Data were analyzed using a one-way ANOVA (Dunnett’s multiple comparison test). All samples were compared with the control (wild type). ****, *P* < 0.0001. ns, not statistically significant.

A previous report showed that mutant spores lacking the SpsA-L proteins are strongly impaired in their germination efficiency (18). To better characterize the role of the glycan produced by the SpsA-L enzymes, purified spores of the *spsA-L* mutant and its isogenic parental strain were compared for their resistance to heat, UV, H_2_O_2_, organic solvents, and trypsin treatment. No differences in viability were observed between wild-type and mutant spores after heat treatment, suggesting that the cortex was normally structured in the mutant spores (Fig. 1A). Wild-type spores were slightly more resistant than mutant spores to UV irradiation (Fig. 1B) and considerably more resistant to a trypsin treatment (Fig. 1C), to the exposure to H_2_O_2_ (Fig. 1D), or to organic solvents (Fig. 1E), suggesting that mutant spores have a defective surface (coat and crust). The results of Fig. 1, together with previous results on the efficiency of germination (18), suggest that the glycan synthesized by the SpsA-L enzymes is involved in the integrity of the spore surface and contributes to the resistance and germination properties of mature spores.

### The hydrophobicity of *spsA-L* spores is due to extractable molecules

**Fig 2:**
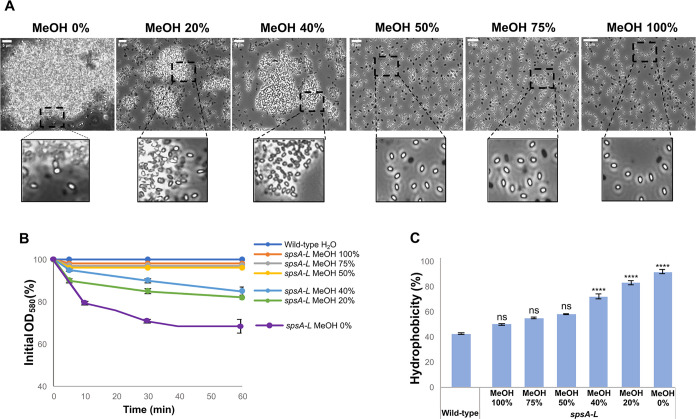
The hydrophobicity of *spsA-L* spores. (A) Optical microscopy fields (100× lens) of *spsA-L* spores suspended at different concentrations of MeOH. Boxes report parts of the same microscopy fields. The size bar is shown in all the principal pictures. (**B**) Clumping assay. Spores of *spsA-L* were suspended in different concentrations of MeOH, and the decrease in optical density (OD_580_) was monitored over time and compared with wild-type suspended in distilled water. The percentage data are averages from three independent experiments performed with spores prepared independently, and the error bars represent standard deviations. (**C**) BATH assay. The percentage of hydrophobicity of *spsA-L* mutant spores was calculated as previously reported (26). The data are averages from three independent experiments performed with spores prepared independently. Data were analyzed with a one-way ANOVA test (Dunnett’s multiple comparison test). All samples were compared with the control (wild-type). ****, samples considered statistically significant compared with the control with *P* < 0.0001. ns, not statistically significant.

It has been previously reported that *B. subtilis* strains lacking the products of the *spsA-L* operon produce spores that form large aggregates that tend to precipitate in an aqueous solution (18). The formation of such aggregates was suggested to be due to hydrophobic interactions between spores suspended in an aqueous environment (18). To investigate the nature of those interactions, wild-type and *spsA-L* mutant spores were suspended in the presence of methanol (MeOH) and analyzed for clump formation. Under a light microscope equipped with a 100× lens, in similarly crowded microscopy fields, small clumps were observed with 20% and 40% MeOH and no clumps were found with MeOH concentration higher than 40% (Fig. 2A). No clump formation was observed when wild-type spores were suspended in MeOH (Fig. S1A). Aggregates of *spsA-L* spores (1.5 ± 0.1 × 10^8^ spores, corresponding to an OD_580_ of 0.6) suspended in 20% or 40% MeOH tended to precipitate, causing the decrease of the optical density of the spore suspension over time (Fig. 2B). When the spores were suspended in 50%, 75%, or 100% MeOH, a decrease in the optical density was not observed for the entire duration of the experiment (60 min) (Fig. 2B).

The effect of MeOH on wild-type and *spsA-L* spores was also measured using the BATH assay (26). In this assay, the spores suspended in water were vigorously mixed with hexadecane, and the two phases were then allowed to separate. Hydrophobic spores accumulate at the interface between water and the solvent, and therefore, the number of spores remaining in the aqueous phase decreases. This decrease indirectly measures the hydrophobicity of the spore, and its inverse is given as a percentage of relative hydrophobicity (26). As shown in Fig. 2C, *spsA-L* spores suspended in 20% or 40% MeOH were more hydrophobic than isogenic wild-type spores suspended in an aqueous solution (*P* < 0.001). Those suspended in MeOH concentrations higher than 40% were slightly more hydrophobic than wild-type spores suspended in an aqueous solution, but these differences were not statistically significant (Fig. 2C). No hydrophobicity variations were observed when wild-type spores were suspended in MeOH (Fig. S1B).

**Fig 3:**
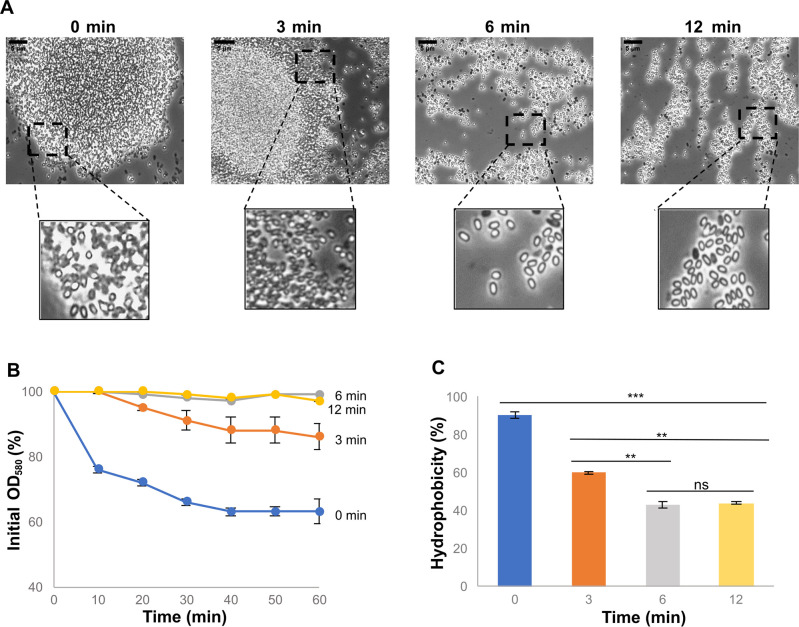
Effect of ultrasonication treatment on *spsA-L* spores. (A) Optical microscopy fields (100× lens) of *spsA-L* spores after ultrasonication treatment at different times with 40% amplitude and energy input 30’’ on and 30’’ off. Boxes report parts of the same microscopy fields. A size bar is shown in all the principal pictures. (**B**) Clumping assay. Spores of *spsA-L* after ultrasonication treatment at different times were monitored, and the decrease in optical density (OD_580_) over time was observed. Error bars represent the standard deviations of three independent experiments performed with spores prepared independently. (**C**) BATH assay after ultrasonication treatment of 0, 3, 6, and 12 min. The data are averages from three independent experiments performed with spores prepared independently. Data were analyzed with a one-way ANOVA test (Tukey’s multiple comparison test). ***, samples were considered statistically significant with *P* < 0.001; **, *P* < 0.01; ns, not statistically significant.

A possible explanation of the results of Fig. 2 is that MeOH extracts some hydrophobic molecules from the surface of *spsA-L* mutant spores, making them less hydrophobic. To support such a hypothesis, the mutant spores (1.5 ± 0.1 × 10^8^) were resuspended in sonication buffer, sonicated at the 40% amplitude level for 3, 6, or 12 min, and observed under a light microscope (with a 100× lens). As shown in Fig. 3A, large clumps were still present after 3 min of sonication but were drastically reduced after 6 and 12 min of treatment. The same conclusion was reached by measuring the optical density of *spsA-L* spores (1.5 ± 0.1 × 10^8^ spores, corresponding to an OD_580_ of 0.6) suspended in water and sonicated various times. Although the decrease in optical density was reduced after 3 min of sonication, it was abolished after 6 and 12 min (Fig. 3B), suggesting that the molecule responsible for the hydrophobic interactions between *spsA-L* spores was extracted by sonication. The results of a BATH assay confirmed that the *spsA-L* spore hydrophobicity decreased with the increase in the length of sonication time (Fig. 3C).

**Fig 4:**
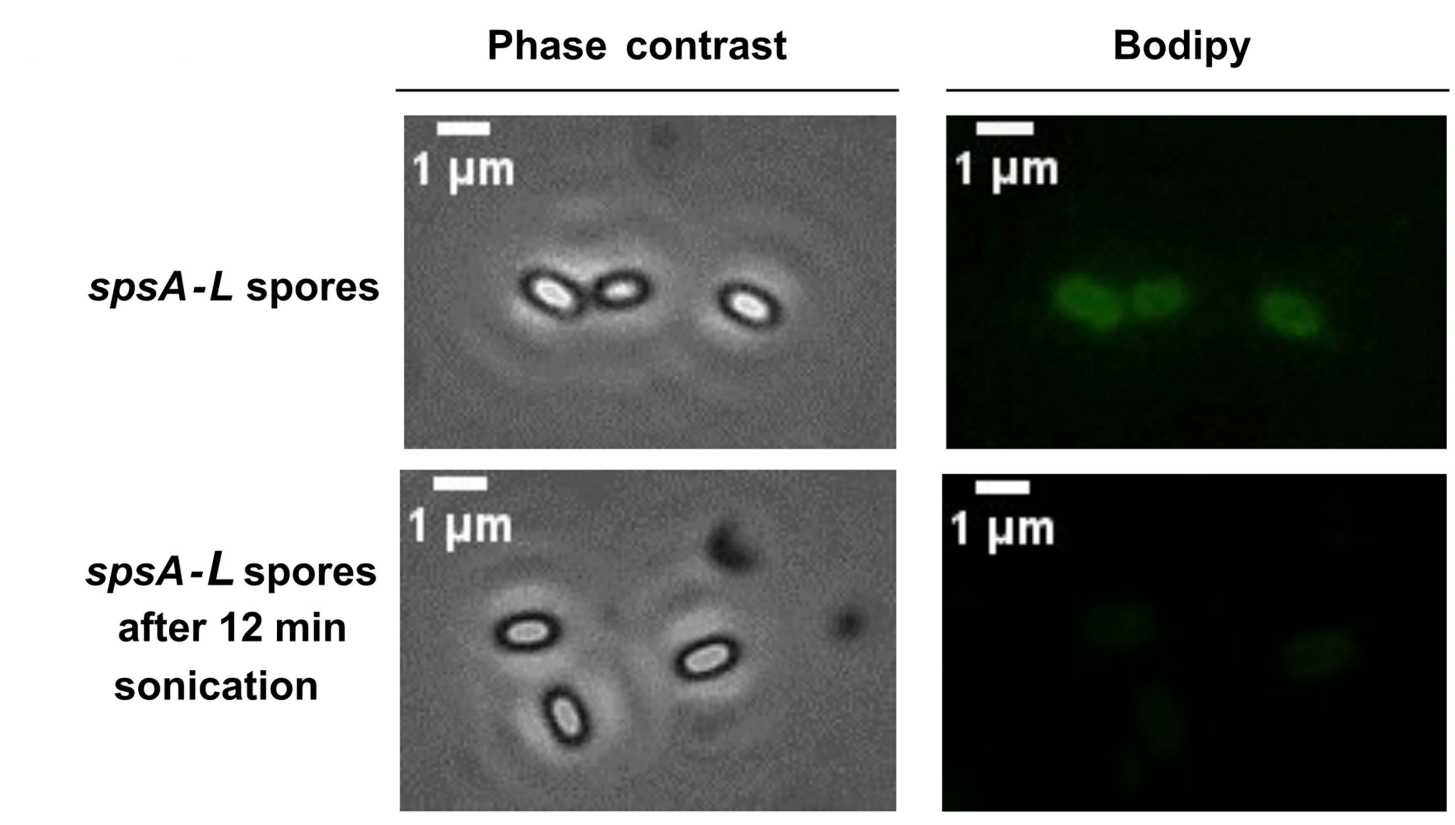
Fluorescence detection before and after ultrasonication treatment. Fluorescence microscopy analysis of mutant spores *spsA-L* (1.5 ± 0.1 × 10^8^) stained with BODIPY at a final concentration of 1 µg/mL before and after ultrasonication treatment. The exposure time was 500 ms for both panels. Scale bar, 1 µm.

The conclusion that hydrophobic molecules were released from the spore following sonication was further confirmed by staining *spsA-L* spores with bodipy (BODIPY 493/503 Thermo Fisher Scientific; dissolved in DMSO and used at a final concentration of 1 µg/mL), a fluorescent stain known to bind hydrophobic molecules, such as neutral or non-polar lipids (27). Spores were stained with bodipy before or after 12 min of sonication as described above and then analyzed by fluorescent microscopy. The fluorescence levels appeared clearly reduced by the sonication, confirming that a hydrophobic molecule present on the surface of *spsA-L* spores was removed by the treatment (Fig. 4).

Taken together, the results of Fig. 2 to 4 confirm that *spsA-L* spores aggregate in an aqueous suspension because of their hydrophobicity and indicate that such effect is due to the surface exposure of hydrophobic molecules that in wild-type spores are most likely covered by a glycan layer.

### Tetraprenyl curcumene is responsible for the hydrophobicity of *spsA-L* spores

**Fig 5:**
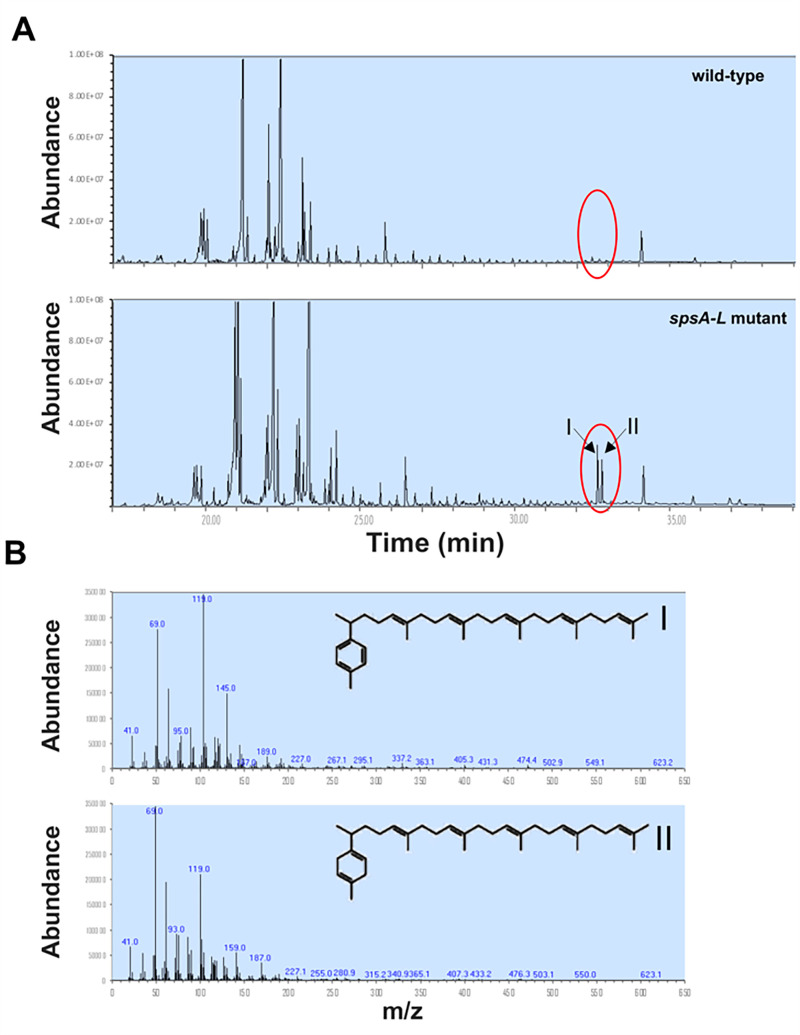
Detection of molecules responsible for the hydrophobicity of *spsA-L* spores. (A) Total ion chromatogram of the lipid extracts of spores of the wild-type (upper panel) and *spsA-L* mutant (lower panel) strains. Ten milligrams of dried spores were suspended in 0.9% sodium chloride solution, and the lipids were extracted with CHCl_3_:MeOH (2:1). Specific internal standards were added to the samples. Time is expressed in minutes of retention. (**B**) Mass spectrum of Peak I (upper panel) and Peak II (lower panel) is indicated with a red circle, respectively. The structures of acyclic C-35 polyprenoid tetraprenyl-α-curcumene (I) and acyclic C-35 polyprenoid tetraprenyl-β-curcumene (II) detected in the spores of *B. subtilis* are reported.

**Fig 6:**
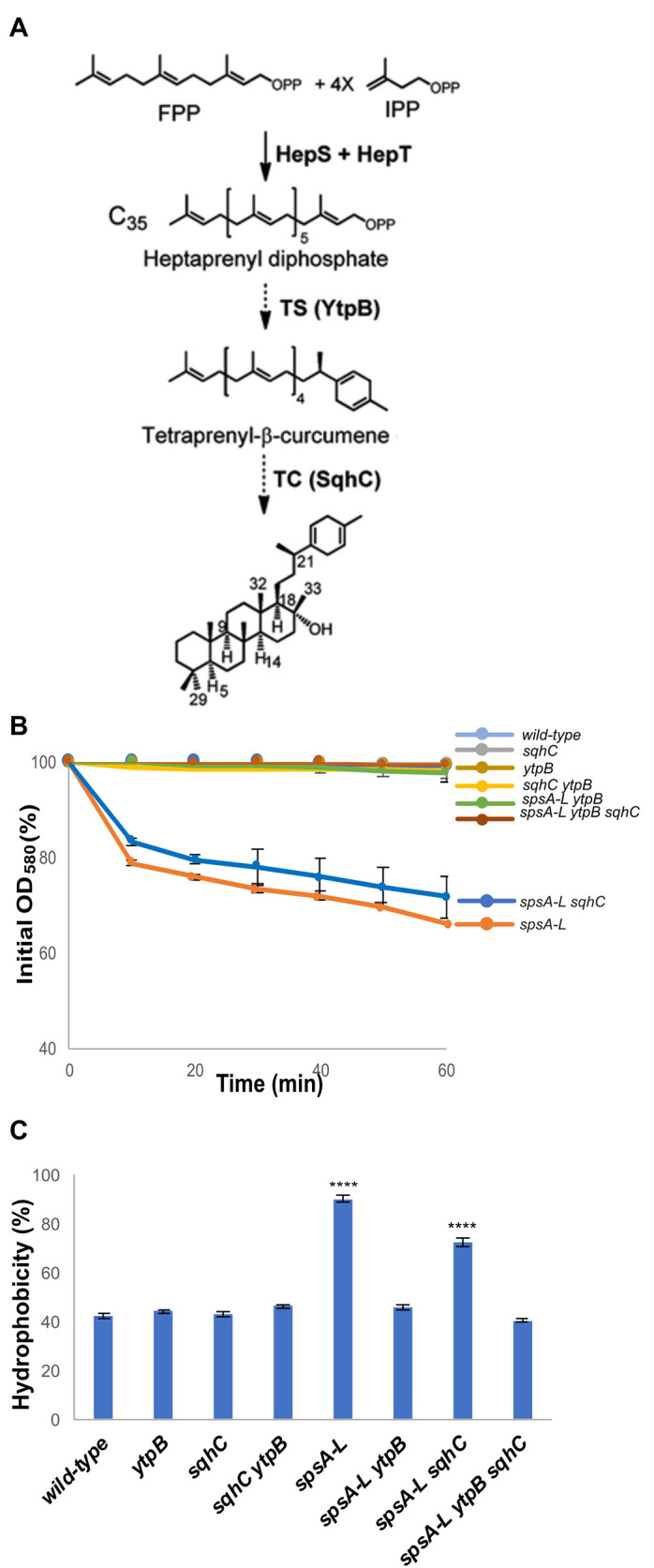
Tetraprenyl curcumene is responsible for *spsA-L* spores hydrophobicity. (A) The tetraprenyl curcumene and sporulene biosynthetic pathway (24). (**B**) Clumping assay of wild-type spores and mutant spores suspended in distilled water. The decrease in optical density (OD_580_) was monitored over time. Error bars represent standard deviations of three independent experiments performed with spores prepared independently. (**C**) BATH assay of wild-type spores and mutant spores. The percentage data are averages from three independent experiments performed with spores prepared independently. Data were analyzed with a one-way ANOVA test (Dunnett’s multiple comparison test). All samples were compared with the control (wild-type). ****, samples considered statistically significant with *P* < 0.0001.

To identify the hydrophobic molecule responsible for the hydrophobic interactions between *spsA-L* spores, 10 mg of dried wild-type and *spsA-L* mutant spores were incubated in a mixture of chloroform and methanol (2:1) for 1 h at room temperature and analyzed by gas-chromatography mass spectrometry (GC-MS) analysis. This mild procedure was chosen to avoid the spores’ lysis and, hence, the extraction of lipids from their deeper layers. In addition to the typical microbial branched-chain and hydroxy-fatty acids with varying chain lengths from C12 to C20, the GC-MS analysis revealed the presence of two peaks (Fig. 5A, peaks I and II) more abundant in the mutant than in the wild-type spores. Peaks I and II were identified by mass spectra analysis as the alpha and beta forms of tetraprenyl curcumene, respectively (Fig. 5B). The same molecules were previously extracted from *B. subtilis* spores (24) and identified as the precursor of sporulene, a spore-associated pentacyclic triterpenoid (24). The tetraprenyl curcumene and sporulene biosynthetic pathway are schematically reported in Fig. 6A (28). The action of two heptaprenyl-diphosphate synthases (HepS and HepT) generates a linear 35-carbon heptaprenyl diphosphate that is converted into tetraprenyl curcumene by the tetraprenyl curcumene synthase YtpB and then into the cyclic sporulene by the cyclase SqhC (Fig. 6A) (28). To confirm that tetraprenyl curcumene and its derivative sporulene were involved in the hydrophobicity of *spsA-L* spores, null mutations in genes coding for the tetraprenyl curcumene biosynthetic enzyme YtpB and for the cyclase SqhC (29) were separately introduced into wild-type or isogenic *spsA-L* mutant strains by chromosomal DNA-mediated transformation. Spores were purified from the resulting strains and analyzed for their hydrophobicity by assessing the decrease of optical density (Fig. 6B) and by the BATH assay (Fig. 6C). Although the optical density of *spsA-L* and *spsA-L sqhC* spores suspended in water decreased over time, that of all other tested strains remained unaltered for the entire duration of the experiment (60 min) (Fig. 6B). Consistently, in a BATH assay, null mutations in *sqhC* or *ytpB* did not affect spore hydrophobicity in a wild-type background but affected the hydrophobicity in *spsA-L* mutant backgrounds (Fig. 6C). In particular, in spores lacking SpsA-L, a null mutation in the *sqhC* gene did not significantly reduce the hydrophobicity that was instead reduced by a null mutation in the *ytpB* gene (Fig. 6C). The obtained results are summarized in Table 1 and allow us to conclude that the molecule responsible for the hydrophobicity of *spsA-L* mutant spores is the tetraprenyl curcumene.

**TABLE 1:** Aggregation tendency of wild-type and mutant spores

Relevant genotype	Clump formation^*a*^
Wild type	–
*ytpB*	–
*sqhC*	–
*ytpB sqhC*	–
*spsA-L*	+
*spsA-L ytpB*	–
*spsA-L sqhC*	+
*spsA-L ytpB sqhC*	–
*cotVWXYZ*	+
cotVWXYZ *spsA-L*	+
*cotVWXYZ sqhC*	+
*cotVWXYZ ytpB sqhC*	–
*cgeA*	+
*cgeA sqhC*	+
*cgeA ytpB sqhC*	–

^*a*^ "+" and "–" indicate that clumps were or were not formed, respectively.

### Tetraprenyl curcumene is not exposed on the surface of wild-type spores

**Fig 7:**
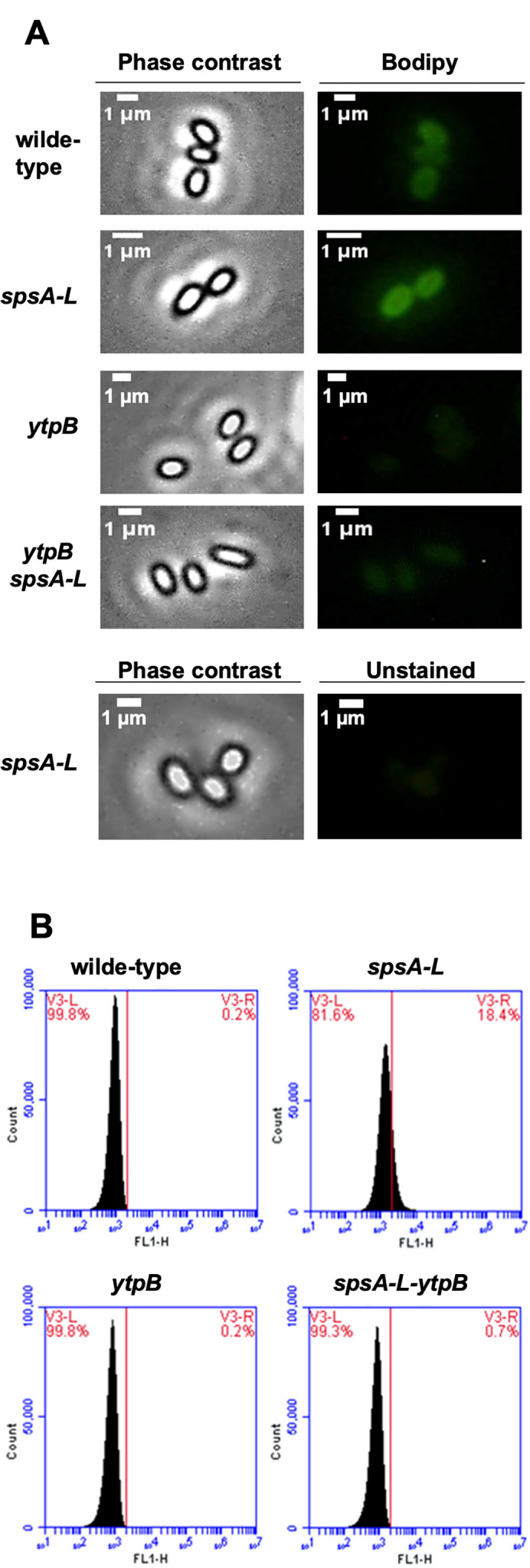
Fluorescence detection. (A) Fluorescence microscopy analysis of wild-type and mutant spores. Spores (1.5 ± 0.1 × 10^8^) were stained with BODIPY at a final concentration of 1 µg/mL. The exposure time was 500 ms for each panel. Scale bar 1 µm. As a control, *spsA-*L mutant spores were observed in the same condition without BODIPY staining. (**B**) Flow cytometry analysis of wild-type and mutant spores. Spores of each sample (1.5 ± 0.1 × 10^8^) were stained with BODIPY (1 µg/mL). In each panel, the percentage of positive events above the fluorescent intensity (right of the red line) and negative events (left of the red line) is indicated.

Wild-type and mutant spores were stained with bodipy (27) and analyzed by cytofluorimetry (30) and fluorescent microscopy, as described above. Specificity of the bodipy staining for tetraprenyl curcumene was indicated by the complete lack of fluorescence signals in mutant spores lacking YtpB (and therefore lacking tetraprenyl curcumene), both in wild-type or in a *spsA-L* background (Fig. 5). Minimal fluorescence levels were associated with wild-type spores, whereas *spsA-L* spores showed high fluorescence signals (Fig. 7A), quantified by the flow cytometer (0.2% of fluorescent wild-type spores vs 18.5% of fluorescent *spsA-L* spores) (Fig. 7B).

Tetraprenyl curcumene, present in wild-type spores (Fig. 5A), is not stained by bodipy (Fig. 7), suggesting that it may be covered by the glycan layer produced by the SpsA-L enzymes. When these enzymes are lacking, tetraprenyl curcumene is surface-exposed, and as a consequence, it is abundantly extractable (Fig. 5A), it is accessible to bodipy staining (Fig. 7), and the mutant spores are highly hydrophobic.

### Tetraprenyl curcumene is responsible for the hydrophobicity of *cotVW-XYZ* and *cgeA* spores

**Fig 8:**
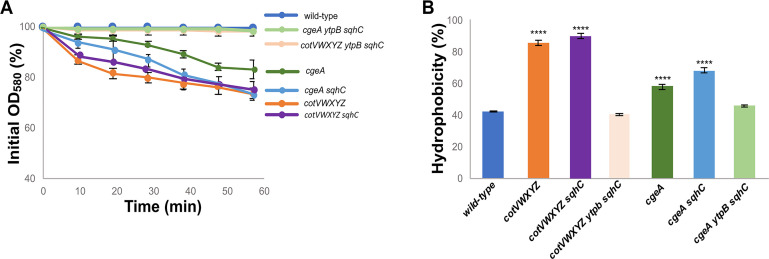
Tetraprenyl curcumene is responsible for *cotVWX-YZ* and *cgeA* spores hydrophobicity. Clumping (**A**) and BATH (**B**) assays of wild-type and *cotVWXYZ*, or *cgeA* mutant spores alone and with deletion of only *sqhC* or of *htpB* and *sqhC*. (A) Purified spores were suspended in distilled water and the decrease in optical density (OD_580_) was monitored over time. Error bars represent standard deviations of three independent experiments performed with spores prepared independently. (B) The percentage data are averages from three independent experiments performed with spores prepared independently. Data were analyzed with a one-way ANOVA test (Dunnett’s multiple comparison test). All samples were compared with the control (wild-type). ****, samples considered statistically significant with *P* < 0.0001.

To evaluate the role of tetraprenyl curcumene in mediating the hydrophobicity of mutant spores lacking the crust proteins CotVWXYZ or CgeA, null mutations in genes coding for the tetraprenyl curcumene biosynthetic enzyme YtpB and the cyclase SqhC (29) were introduced together or separetely into *cotVW-XYZ* or *cgeA* mutant strains by chromosomal DNA-mediated transformation. Spores were purified from the resulting strains and analyzed for their hydrophobicity by assessing the decrease in optical density (Fig. 8A) and by the BATH assay (Fig. 8B). The optical density of a water suspension of *cotVW-XYZ* spores decreased over time (Fig. 8A, orange line), and the spores appeared highly hydrophobic in a BATH assay (Fig. 8B, orange bar). The inactivation of only *sqhC* in the *cotVW-XYZ* mutant did not modify the hydrophobicity of the spores (Fig. 8, purple line and bar), whereas the simultaneous inactivation of *ytpB* and *sqhC* in the *cotVW-XYZ* mutant strongly reduced the spore hydrophobicity (Fig. 8, pink line and bar). *cgeA* mutant spores are less hydrophobic than *cotVW-XYZ* spores, the optical density of a water suspension of *cgeA* spores slowly decreased over time (Fig. 8A, dark green line), and the spores appeared weakly hydrophobic in a BATH assay (Fig. 8B, dark green bar). The inactivation of only *sqhC* in the *cgeA* mutant slightly increased the hydrophobicity of the spores (Fig. 8, light blue line and bar), whereas the simultaneous inactivation of *htpB* and *sqhC* in the *cgeA* mutant strongly reduced the spore hydrophobicity (Fig. 8, light green line and bar).

Results of Fig. 8 indicate that tetraprenyl curcumene is responsible for the hydrophobicity of *cotVW-XYZ* or *cgeA* mutant spores. The observation that the *cgeA* spores were weakly hydrophobic suggests that the triterpenoid was still partially covered by glycans and only partially exposed on the surface of *cgeA* spores.

## DISCUSSION

At least four sporulation gene clusters, *spsABCDEFGHIJKL*, *yfnHGF-yfnED*, *ytdA-ytcABC*, and *cgeAB-cgeCD*, have been identified as responsible for the synthesis of the spore surface polysaccharides (14, 15). In mutants lacking the products of each of these operons, the morphology and the properties of the spore surface are altered (15). A mutant lacking the *spsA-L* operon produces highly hydrophobic (14, 18) and germination-defective (18) spores that are also less resistant than spores of the isogenic wild-type strain (Fig. 1). This study demonstrates that the hydrophobicity of *spsA-L* mutant spores is due to the exposure of a hydrophobic molecule identified by GC-MS and genetic analysis as a sesquiterpenoid compound, the tetraprenyl curcumene.

Tetraprenyl curcumene is synthesized by YtpB, a tetraprenyl curcumene synthase, whose structural gene is part of the *ytpAB* operon, expressed by the alternative sigma factors σ^M^ under stress conditions (31) or σ^E^ during sporulation (32). As a secondary metabolite, tetraprenyl curcumene has a potential role in modulating *B. subtilis* cell integrity and bacitracin resistance (31) and serves as a precursor for the squalene cyclase (SqhC)-catalyzed synthesis of sporulene, a spore-associated pentacyclic lipid (24). The results of this study indicate that when YtpB was not present, and, therefore, tetraprenyl curcumene was not produced, *spsA-L* spores were no longer highly hydrophobic, clearly indicating that tetraprenyl curcumene and/or its derivative sporulene are the molecule responsible for the hydrophobicity of *spsA-L* spores. When SqhC was not present, and, therefore, tetraprenyl curcumene was not converted into sporulene, *spsA-L* spores were still highly hydrophobic, indicating that the role of sporulene in mediating the hydrophobicity of *spsA-L* spores is minimal. This conclusion is somehow surprising since tetraprenyl curcumene and its cyclic derivative sporulene should have a similar hydrophobicity. A simple explanation for this unexpected result is that the experimental approaches used in this study to measure spore hydrophobicity are not really quantitative and failed to detect differences in the hydrophobicity of *spsA-L ytpB* vs. *spsA-L sqhC* spores. An alternative explanation is that only a minimal amount of tetraprenyl curcumene is converted into sporulene on the spore surface; therefore, the amount of sporulene on the spore surface is very low, and its contribution to the hydrophobicity of *spsA-L* spores is minimal. Support for this alternative explanation comes from the observation that sporulene could not be detected by GC-MS analysis in this study, and it was detected on the spore surface only when its coding gene, *sqhC*, was over-expressed (24). In this context, it is interesting to note that in the weakly hydrophobic *cgeA* spores, the deletion of the *sqhC* gene increased the hydrophobicity (Fig. 8). A possible explanation is that without the squalene cyclase (SqhC), converting tetraprenyl curcumene into sporulene, the substrate of the enzyme accumulates and increases the total hydrophobicity of the spore. An alternative explanation is that the lack of SqhC, previously localized in the spore coat (24), alters the coat structure, thus affecting the interaction between coat proteins and spore surface glycans, the exposure of the triterpenoid, and, as a consequence, the spore hydrophobicity. Further studies will be needed to fully elicit this point.

**Fig 9:**
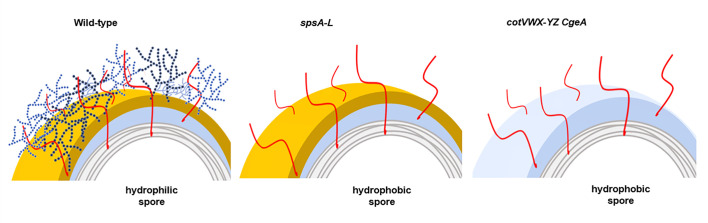
Working model. Schematic representation of the spore surface organization of wild-type and mutant spores. The crust is indicated in yellow, the outer coat in light blue, the inner coat in light gray, the *spsA-L*-dependent polysaccharides in dark blue, and the tetraprenyl curcumene in red. The anchoring site of tetraprenyl curcumene is still unknown, and in the present model, the molecule is shown to originate from either the outer or inner coat.

It has been previously shown that spores of mutant strains lacking the *cotVW-XYZ* or *cgeA* genes are highly hydrophobic (12). It is shown here that tetraprenyl curcumene is the molecule responsible for the observed spore hydrophobicity also in these cases. These results then allow us to propose that tetraprenyl curcumene is present on the *B. subtilis* spore surface, where it is covered by polysaccharides produced by the action of at least some of the SpsA-L enzymes bound to some of the crust proteins (Fig. 9). When these polysaccharides (dark blue in Fig. 9) are not synthesized (as in *spsA-L* mutant spores) or when their glycosylation targets are not present (as in *cotVWX-YZ* or *cgeA* mutants), tetraprenyl curcumene (red in Fig. 9) is surface exposed, making the spore highly hydrophobic. A previous study has reported that spores of a mutant lacking only the last four genes of the *spsA-L* operon (*spsIJKL*), responsible for the synthesis of rhamnose, are highly hydrophobic like those lacking the entire operon (15). Therefore, it is likely that rhamnose is attached to a crust protein to start the polysaccharide layer that covers the tetraprenyl curcumene in wild-type spores (Fig. 9). The triterpenoid exposed on the spore surface is certainly not anchored to the crust, since spores lacking the crust are still highly hydrophobic. The attachment site of the tetraprenyl curcumene is at the moment not known, and its identification is a challenging goal of future studies.

## MATERIALS AND METHODS

### Bacterial strains and mutant construction

Bacterial strains used in this study are listed in Table S1. To obtain null mutations in the *cotVWX* and *cotYZ* operons, two genomic fragments of 801 bp and 817 bp, containing parts of the *yjcA* gene (adjacent to the 5’ end of the *cotV* gene on the *B. subtilis* chromosome) and the *cotZ* gene, respectively, were PCR amplified using the *B. subtilis* chromosome as a template and oligonucleotides VWXYZ sense/VWXYZ anti and POST Z sense/POST Z anti (Table S2). The PCR products were cloned into the pGEM-T Easy vector (Promega), yielding plasmids pPreV and pPostZ. Next, the *Bam*HI-*Sph*I fragment from pPreV and the *Bgl*II-*Sal*I fragment from pPostZ were excised and cloned in two steps, upstream and downstream of the neomycin resistance gene in the vector pAH250 (33), which had been previously digested with the appropriate restriction enzymes. The resulting plasmid, pTS37, was used to transform competent cells of the PY79 strain. A double crossover event between the chromosomal DNA of strain PY79 and of the plasmid allowed the replacement of the *cotVWX* and *cotYZ* operons with neomycin-cassettes and the construction of the null mutant. Strain RH316 was obtained by transforming strain GC355 (*sps::neo*) with a linearized form of plasmid pVK71 (34) to inactivate neo resistance and introduce a spectinomycin resistance gene. Several clones resistant to spectinomycin but sensitive to neomycin were isolated, and one of them, RH316, was used for further studies. Moreover, the neo^r^ determinant (neo) of strain GC355 (*spsA-L::neo*) was replaced with a spectinomycin resistance gene cassette (spec) by using plasmid pECE140 (Bacillus Genetic Stock Center, Columbus, OH) originating RH316 strain.

Chromosomal DNA of *B. subtilis* RH300 (*cotVWXYZ::spec*), HB13350 (*ytpB::spc*), HB13358 (s*qhC::MLS*), and HB13360 (*sqhC::MLS; ytpB::spec*) was used to transform competent cells of GC355 (*spsA-L::neo*), RH316 (*spsA-L::spec*), and GC347 (*cgeA::spc*) generating strains reported inTable S1.

### Sporulation and spore purification

Sporulation was induced in the Difco sporulation (DS) medium by the exhaustion method (35). After 30 h of incubation at 37°C ± 1, the spores were collected, washed four times, and incubated in H_2_O at 4°C overnight to lyse residual sporangial cells (36). The spores were washed with cold H_2_O several times until they were 95% pure by microscopic inspection. All microscopy images were captured using light microscopy (Olympus BX51) with 100× magnification and Olympus DP70 digital camera equipped with an Olympus U-CA magnification changer and processed with Image Analysis software (Olympus) for minor adjustments of brightness, contrast, and color balance images.

### Spore resistance properties

Spore resistance was tested as previously reported by (35), with some modifications. For each treatment, water-purified spores were used at final concentrations of 1.5 ± 0.1 × 10^8^ (corresponding to an OD_580_ of 0.6). After each treatment, the spore resistance was observed by serial dilutions on TY agar plates and counting the colony-forming units (CFU) per mL of culture after overnight incubation at 37°C. The percentage of resistance is given by the formula (spores treated/spores untreated) × 100.

For heat resistance, heat-treated (80°C for 10 min) and untreated spores were serially diluted in PBS 1× buffer and plated on TY agar. For ultraviolet (UV) light resistance, spores were suspended in a sterile glass 10 cm diameter petri dish and irradiated with a UV lamp (700 erg/mm^2^) set at a predetermined distance from the suspension (20 cm). Before irradiation and at 3 and 5 min after the beginning of irradiation, the spores were serially diluted in PBS 1× buffer and plated on TY agar. For trypsin resistance, spores were incubated with trypsin (neoFroxx, Germany) at a final concentration of 0.1 mg/mL for 1 h at 37°C. Untreated and treated spores were serially diluted in PBS 1× buffer and plated on TY agar. For hydrogen peroxide (H_2_O_2_), spores were incubated with 5% H_2_O_2_ (Sigma-Aldrich) at room temperature for 5, 15, and 30 min. At various time points, the spores were serially diluted in PBS 1× buffer and plated on TY agar. For organic solvent resistance, the spores were incubated at room temperature for 10 min with pure chloroform (CHCl_3_) or toluene (C_6_H_5_CH_3_) (Sigma-Aldrich), adding 100 µL of solvent in a final volume of 1 mL. Treated and untreated spores were serially diluted in PBS 1× buffer and plated on TY agar. The experiments were three replicated with spores prepared independently.

### Methanol and ultrasonication treatment

Mutant spores (1.5 ± 0.1 × 10^8^ spores) suspended in distilled water were centrifuged for 10 min at 7,000 × *g* and then suspended in 2 mL of methanol (MeOH) (Sigma-Aldrich) at different concentrations (0%, 20%, 40%, 50%, 75%, and 100%). The effect of MeOH was observed under light microscopy (Olympus BX51) with 100× magnification. For ultrasonication treatment, 1.5 ± 0.1 × 10^8^ *spsA-L* spores were centrifuged at 7,000 × *g* for 10 min and suspended in a sonication buffer (50 mM Tris-HCl [pH 6.8], 1 mM PMSF, 50 mM EDTA [pH 8], 5 mM DTT, 20% MeOH). The treatment was carried out with a constant amplitude of 40% for 0, 3, 6, and 12 min with energy input at 30 s on and 30 s off using a medium power tip sonicator (Sonics, Vibra cell VC505). During the sonication treatment, the spores were kept in an ice-cold bath. At the end of ultrasonication treatment, the *spsA-L* spores were centrifuged at 7,000 × *g* for 10 min to discard the supernatant, washed in distilled water for trice time, and observed under light microscopy (Olympus BX51) with 100× magnification. Both experiments were repeated three times.

### Clumping and BATH assays

In the clumping assay, spores (1.5 ± 0.1 × 10^8^ spores, corresponding to an OD_580_ of 0.6) were counted under an optical microscope (Leica DM750, 40 × magnification) with a Burker chamber and suspended in distilled water in a cuvette. The samples were vigorously mixed and placed in the spectrophotometer (UV-5600), and the OD_580_ was measured every 10 min for 1 h (17). The spores were suspended in MeOH at different concentrations (0%, 20%, 40%, 50%, 75%, and 100%) instead of distilled water to test the effect of MeOH. After ultrasonication treatment, the spores were centrifuged at 7,000 × *g* for 10 min to remove the buffer sonication, and then, they were suspended in distilled water to test the clumping assay.

For the BATH assay (26), 3.0 mL of each spore suspension (1.5 ± 0.1 × 10^8^ counted under an optical microscope with a Burker chamber) were incubated for 15 min at 25°C ± 1 with 1.0 mL of hexadecane (Sigma-Aldrich). The mixture was vortexed for 1 min in glass test tubes (15 by 100 mm), and the hexadecane and aqueous phases were allowed to partition for 15 min. The aqueous phase was carefully removed using a Pasteur pipette, and the OD_440_ was measured. As previously reported (26), the decrease in OD_440_ of the aqueous suspension indicated the relative hydrophobicity, and this was calculated as 100(A0 − Af)/A0, where A0 and Af were the initial and final OD_440_, respectively.

Also, in this case, after treatment with MeOH and ultrasonication, the spores were suspended in water, and the assay was performed as reported above. Three independent experiments replicated trice, which were performed with spores prepared independently.

### Extraction of lipids

Lipid extraction from spores for GC-MS analysis was performed following the Folch method with some modifications (37). Purified wild-type and mutant spores were centrifuged at 10,000 × *g* for 5 min, and the supernatant was discarded. The centrifugation was repeated until no supernatant was separated. Ten-milligram aliquots of dry spores were resuspended in 200 µL of 0.9% NaCl and spiked with a mixture of internal standards (60 µg triheptadecanoate; 60 µg heptadecanoic acid). Then, 800 µL of chloroform:methanol (2:1. vol/vol) mixture was added, and the samples were incubated for 1 h at room temperature under shaking. After centrifugation (10,000 × *g* for 5 min), the lower organic layer was separated and divided to conduct different derivatization processes to analyze not only fatty acids but also other polar components in the extracts.

### Transesterification with sodium methoxide (NaOMe)

The composition and content of bound and free fatty acids in the samples (200 µL lipid extract evaporated into dryness) were determined after transesterification with 500 µL of 0.5 N sodium methoxide (Sigma-Aldrich) and boiling at 45°C for 5 min (38, 39). The samples were acidified with 15% NaHSO_4_ and extracted with 1 mL of petroleum ether (Merck–Millipore) at 40–60°C. After centrifugation, the petroleum ether layer was separated and evaporated, and the residue was dissolved into 1 mL of n-hexane. Bound fatty acids were transesterified to methyl esters, whereas free fatty acids remained free. One microliter of the hexane phase was injected in splitless mode into Agilent GC-MS.

### Trimethylsilylation

Free polar groups, like hydroxyl and carboxyl groups, were trimethylsilylated to improve their volatility and stability (40). Aliquots of lipid extracts (100 µL) or methylated samples (evaporated into dryness) were mixed with 50 µL of 1-(trimethylsilyl)imidazole-pyridine mixture (Merck) with 50 µL N-methyl-N-(trimethylsilyl)trifluoroacetamide with 1% trimethylchlorosilane (Merck) at 70°C for 1 h.

### Gas-chromatography mass spectrometry

Fatty acid methyl esters, obtained after HCl-MeOH or NaOMe derivatization, were analyzed by GC-MS (41). The transport gas was helium, with a constant flow rate of 1.2 mL/min. The injector temperature was 240°C, and the splitless mode was used. An HP-FFAP column (25 m × 0.25 mm × 0.33 µm) was used with a temperature program from 40°C (1.5 min) to 240°C (15°C/min, stay for 9 min or 26 min in case free fatty acids were present). The interface temperature was 240°C, and the ion source temperature was 150°C. The mass spectra were recorded over a 35–600 atomic mass unit range at three scans/s. Gas chromatographic analysis of trimethylsilylated samples was performed similarly as described (24). The samples were run on an Agilent 6890 gas chromatograph (GC) combined with an Agilent 5973 mass selective detector (MSD) using an Agilent DB-5MS capillary column (30 m × 0.25 mm × 0.25 µm) and oven temperature program from 40°C (3 min) to 330°C (18 min) at a rate of 10°C/min (run time 50 min). The injection volume was 1 µL, and the injection mode was splitless (Injector temp 260°C). The identification of the compounds was based on the spectral search from the NIST library and the literature (24).

### Surface lipid detection

Water-purified spores (1.5 ± 0.1 × 10^8^) were centrifuged at 7,000 × *g* for 10 min and suspended in 1 mL of 1× phosphate-buffered saline (PBS; pH 7.0) and with a fluorophore BODIPY (BODIPY 493/503 Thermo Fisher Scientific) dissolved in DMSO and used at a final concentration of 1 µg/mL that emits green fluorescence. After 30 min of incubation in darkness, the spores of each sample were analyzed with a flow cytometer (BD Accuri C6; BD Biosciences, San Jose, CA) (42). Data were collected by Count and FLH-1(533/30 nm filter like FITC) gating. Fluorescence analyses were also conducted using an Olympus BX51 fluorescence microscope fitted with a 100× objective UPlanF1.

Fluorescein isothiocyanate (FITC) (U-MNIB2) filters were used to detect the green fluorescence signals (43). Images were captured using an Olympus DP70 digital camera equipped with an Olympus U-CA magnification changer and processed with Image Analysis software (Olympus) for minor brightness, contrast, and color balance adjustments. Fluorescence intensities were measured using ImageJ processing software (version 1.48; NIH). Spores without fluorophore staining were used as a control.

### Statistical analysis

The statistical analyses were performed using GraphPad Prism 8 software (44). Data were expressed as means ± SD. Differences among groups were compared by one-way ANOVA (Dunnett’s multiple comparisons test or Tukey’s multiple comparison test) as indicated in figure legends. Differences were considered statistically significant at *P* ≤ 0.05.

## SUPPLEMENTAL MATERIAL

Figure S1 mbio.03024-24-s0001.tiff Hydrophobicity of wild-type spores.

Tables S1 and S2 mbio.03024-24-s0002.docx *B. subtilis* strains used in this study, and oligonucleotides used to prime amplification reactions.

ASM does not own the copyrights to Supplemental Material that may be linked to, or accessed through, an article. The authors have granted ASM a non-exclusive, world-wide license to publish the Supplemental Material files. Please contact the corresponding author directly for reuse.
