# Supplementary figures and images for: The triterpenoid curcumene mediates the relative hydrophilicity of Bacillus subtilis spores
Source: mBio. 2024 Nov 29;16(1):e03024-24. doi: 10.1128/mbio.03024-24 (PMC11708026; doi:10.1128/mbio.03024-24)

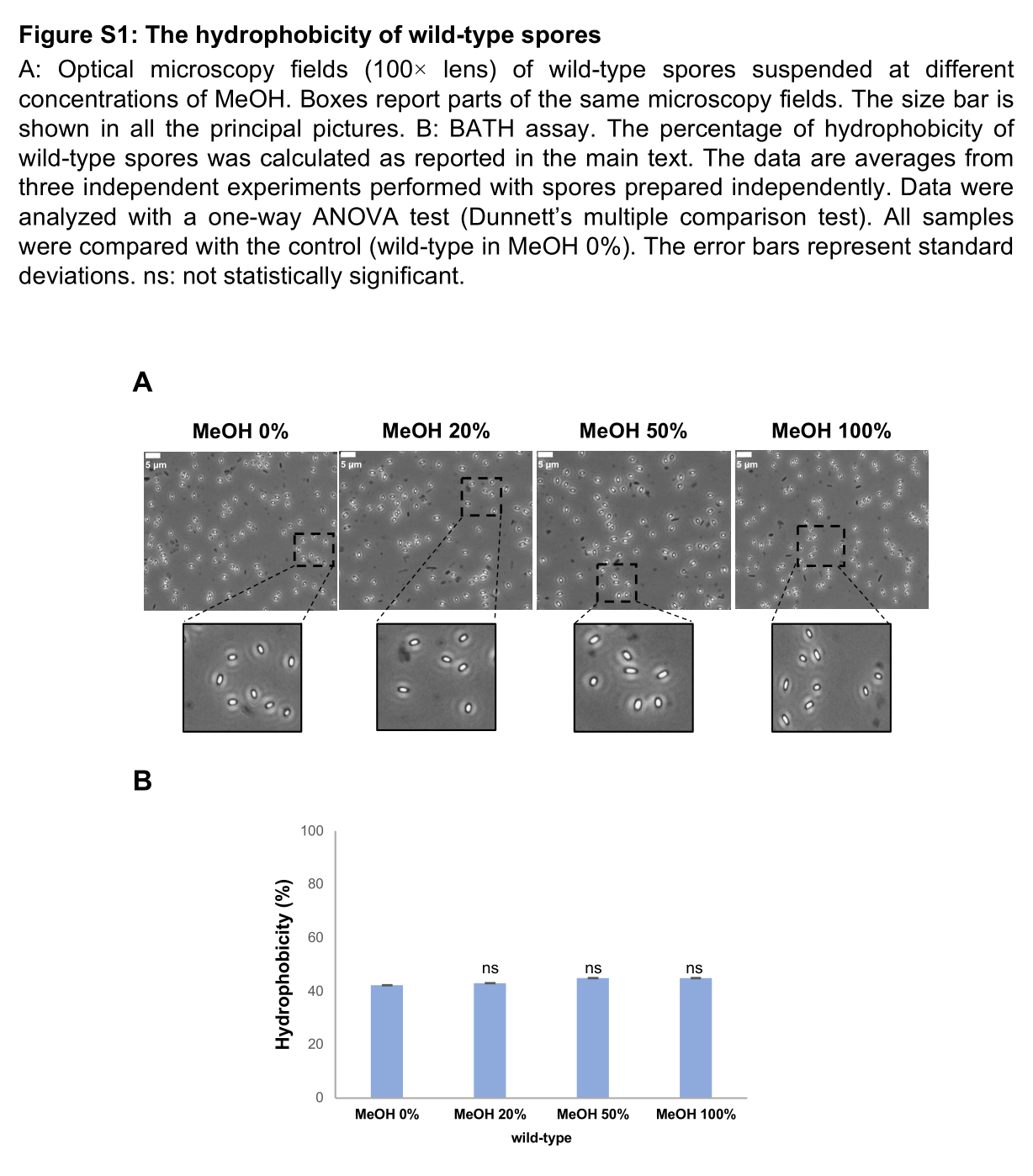

Supplement: Figure S1 — Hydrophobicity of wild-type spores. [file mbio.03024-24-s0001.tiff]
