# Supplementary material for: The triterpenoid curcumene mediates the relative hydrophilicity of Bacillus subtilis spores
Source: mBio. 2024 Nov 29;16(1):e03024-24. doi: 10.1128/mbio.03024-24 (PMC11708026; doi:10.1128/mbio.03024-24)
Supplement: Tables S1 and S2 — B. subtilis strains used in this study, and oligonucleotides used to prime amplification reactions. [file mbio.03024-24-s0002.docx]

**Supplementary Material**

**Table S1. *B. subtilis* strains used in this study**

| Strain | Relevant genotype | Reference or source |
| --- | --- | --- |
| PY79 | wild-type | (2) |
| GC355 | *spsA-L::neo* | (17) |
| GC347 | *cgeA::spc* | (46) |
| RH316 | *spsA-L::spc* | This study |
| RH300 | *cotVWXYZ:: neo* | This study |
| RH301 | *spsA-L::spec cotVWXYZ:: neo* | This study |
| HB13350 | *ytpB::spec* | (28) |
| HB13358 | *sqhC:: MLS* | (28) |
| HB13360 | *sqhC::MLS ytpB::spec* | (28) |
| RH317 | *spsA-L::neo ytpB::spec* | This study |
| RH318 | *spsA-L::neo sqhC:: MLS* | This study |
| RH319 | *spsA-L::neo ytpB::spec sqhC:: MLS* | This study |
| RHF320 | *cgeA::spec ytpB::spec sqhC:: MLS* | This study |
| RHF323 | *cotVWXYZ:: neo ytpB::spec sqhC::MLS* | This study |
| RHF324 | *cotVWXYZ:: neo sqhC::MLS* | This study |
| RHF325 | *cgeA::spec sqhC:: MLS* | This study |
| *neo: neomycin (neo; 5 µg ml^−1^); spec: spectinomycin (spec; 50 µg ml^−1^); MLS: contains 1 µg ml^−1^ erythromycin and 25 µg ml^−1^ lincomycin.* | | |

**Table S2. List of oligonucleotides used to prime amplification reactions**.

| **Primer** | **Sequence** *^a^* | **Restriction site** |
| --- | --- | --- |
| POST Z senso | agatctGCTGTTGAAGAAGACTGC | *BglII* |
| POST Z anti | gtcgacGGAAGAGCCTGCACTTG | *SalI* |
| VWXYZ senso | ggatccGTGGTTCATATCTTCCTTC | *BamHI* |
| VWXYZ anti | gcatgcAAATGGGGTGCAGAGCGG | *SphI* |
| *^a^ Not capital letters indicate restriction recognition sites.* | | |
